# Rebalancing the non-coding RNA research agenda: future directions for long non-coding RNA and circular RNA research

**DOI:** 10.1038/s12276-026-01832-x

**Published:** 2026-09-07

**Authors:** Young-Kook Kim, Hyun Kook

**Affiliations:** 1 Department of Biochemistry, Chonnam National University Medical School, Hwasun, Jeonnam-Gwangju Special Metropolitan City 58128 Republic of Korea; 2 Medical Research Center for Innovative Control of Cardiovascular Remodeling Diseases, Hwasun, Jeonnam-Gwangju Special Metropolitan City 58128 Republic of Korea; 3 Department of Pharmacology, Chonnam National University Medical School, Hwasun, Jeonnam-Gwangju Special Metropolitan City 58128 Republic of Korea

**Keywords:** Long non-coding RNAs, Gene regulation

Long non-coding RNA (lncRNA) and circular RNA (circRNA) biology has changed beyond recognition over the past 20 years. LncRNAs, once dismissed as transcriptional noise, are now accepted as regulators of chromatin, transcription, RNA processing and translation^1^. CircRNAs have moved from a curiosity to a class of molecules with their own back-splicing biology and an early position in clinical development^2^. The number of annotated lncRNA genes in the human genome already exceeds the number of protein-coding genes^3^, and lncRNA- and circRNA-based therapeutics are now in clinical pipelines. *Experimental & Molecular Medicine* (EMM) has tracked this growth closely, and our 2024 Special Feature on regulatory RNA reflected the directions we then considered most promising^4^.

Much of the literature on these molecules being published today, however, follows a strikingly uniform template. A new lncRNA or circRNA is reported as differentially expressed in a disease. Knockdown produces a phenotype in one or two cell lines, the result is confirmed in an animal model, and a small patient cohort is shown to express the RNA in the expected direction. A microRNA (miRNA) target is predicted bioinformatically. A luciferase assay confirms the predicted interaction. The proposed mechanism is competitive endogenous RNA (ceRNA) action, or miRNA sponging. Variations on this template fill the literature week after week, and the same general shape applies to papers showing that an lncRNA binds some protein partner without quantitative or structural evidence that the interaction matters in vivo. A smaller stream of papers describes peptides translated from lncRNAs or circRNAs, which we view as an interesting exception rather than as a defining feature of this class. A retrospective analysis of 35 lncRNA and circRNA research papers published in EMM over the past decade (June 2016 to May 2026, Table 1) illustrates this pattern, with 89% focused on ceRNA or descriptive lncRNA–protein binding mechanisms and only one addressing any of the five underexplored areas discussed below. The ceRNA hypothesis was challenged on quantitative grounds more than 10 years ago^5,6^. Many lncRNAs are present at copy numbers far too low to titrate cellular miRNA pools meaningfully^7^. Yet this template has remained remarkably common.

**Table 1:** Mechanistic focus of long non-coding RNA and circular RNA papers published in *Experimental Molecular Medicine*, June 2016 to May 2026.

Category	*N*	Section
**Saturated paradigms**	**31**	
ceRNA/miRNA sponging	15	Introduction
lncRNA–protein binding (descriptive)	16	Introduction
**Underexplored areas**	**1**	
Upstream regulation of lncRNAs	1	Section 1
Biogenesis and host gene relationship of circRNAs	0	Section 2
Structure and function	0	Section 3
Quantitative biology	0	Section 4
Isoform-specific function	0	Section 5
**Other**	**3**	
**Total**	**35**	

Categorization is based on the primary mechanism proposed in each paper. Some papers fitting more than one category were assigned to the most prominent claim. A total of 35 long non-coding RNA (lncRNA)- and circular RNA (circRNA)-related research papers published in *Experimental Molecular Medicine* during this 10-year period were identified. The “Other” category comprises three papers: one lacking a mechanistic study, one on an messenger RNA-targeting circRNA, and one on peptide coding from a circRNA. Cross-references in the rightmost column indicate the section of this Editorial in which the corresponding underexplored area is discussed.

We are not saying that these mechanisms are wrong. The principles of ceRNA– and lncRNA–protein interactions are real biological phenomena that have advanced this field. Our concern is not with the ceRNA concept itself, but with how often it is invoked as a default mechanism without sufficient quantitative or mechanistic support. A study that repeats this familiar flow under a new RNA name, even with the now standard combination of cell line, animal and patient sample data, adds relatively little that is conceptually new. Multi-system validation has become the baseline expectation rather than a mark of distinction. For research to be truly novel, more is required than ever before. Nevertheless, most papers still follow the established framework.

This Editorial sets out where we think the most productive open questions in lncRNA and circRNA biology are likely to lie (Fig. 1). What follows is not a complete map of the field. It is five areas that we have come to view as particularly underexplored. We offer them as examples to stimulate discussion, not as a fixed or exhaustive agenda. Other emerging directions that share the same change in perspective are equally worth pursuing, and we expect that some of the most interesting work over the coming years will fall outside the five areas listed here.

**Fig. 1:**
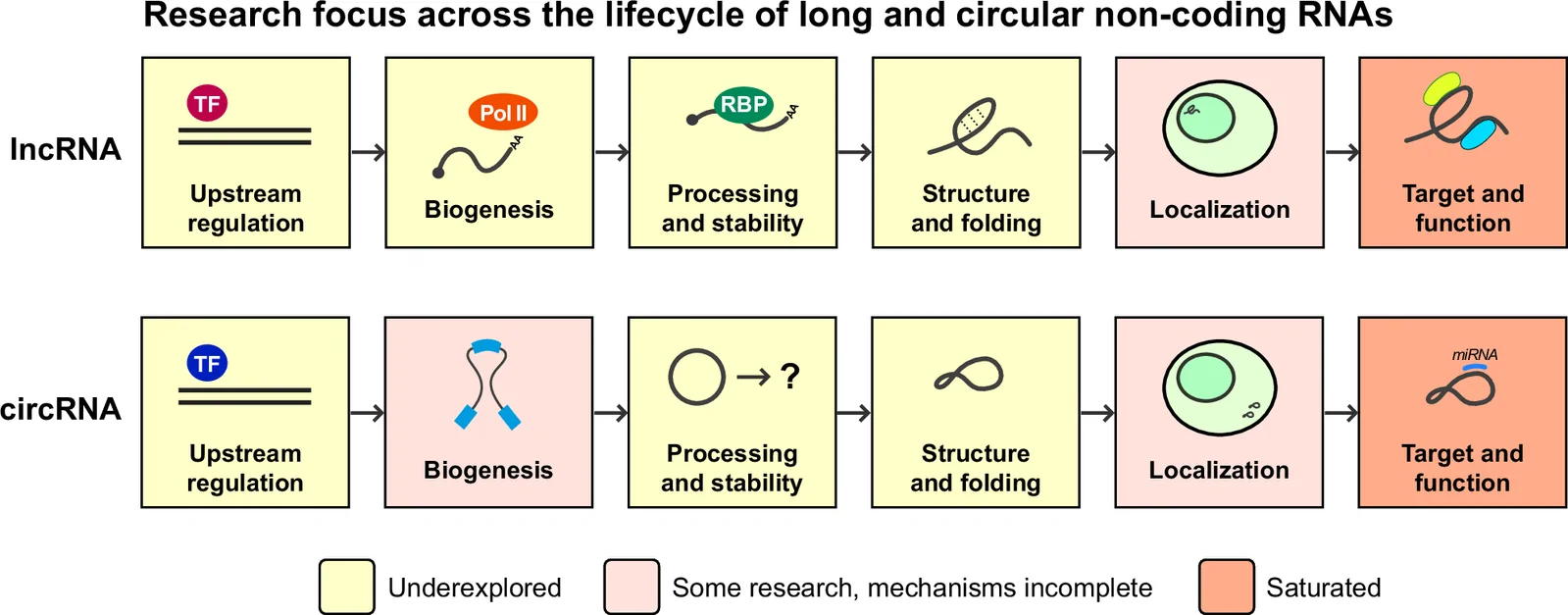
Research focus across the long and circular non-coding RNAs lifecycle. For both long non-coding RNAs (lncRNAs) and circular RNAs (circRNAs), the field has concentrated heavily on the final stage (targets and function) while leaving most upstream stages, including transcriptional regulation, processing, structural folding and stability, comparatively underexplored. The categories shown (underexplored, some research with incomplete mechanisms and saturated) are intended as a conceptual representation of current research trends rather than as a formal, literature-based quantification. The areas highlighted in this figure are illustrative rather than exhaustive, and other emerging directions that share the same shift towards the intrinsic biology of these molecules are equally worth pursuing.

For the past decade, research on these molecules has mostly asked what these molecules do to other molecules. The more productive question for the next decade, in our view, is about the lncRNA or circRNA itself. What controls its expression, what shape it takes, how it is produced, how much of it is actually present in a cell, and which exact isoform we are even talking about. What unites these five areas is a single shift in perspective. The lncRNA or circRNA itself, not just what it does to other molecules, deserves to be the focus. This shift is not only of biochemical interest. How these molecular properties change in disease, what they reveal in patient samples and whether they can be therapeutically modulated are the questions that ultimately determine the clinical relevance of this field.

## Section 1: Upstream regulation of long non-coding RNAs

There is a clear asymmetry in the lncRNA literature. Many papers describe what a lncRNA regulates. Very few ask what regulates the lncRNA itself. Genome-wide analyses have shown that lncRNA promoters carry distinct transcription factor binding patterns, which implies that lncRNAs operate under their own regulatory programme^8,9^. For most disease-associated lncRNAs, however, we still do not know which transcription factors actually drive them, which signals turn them on or why their expression is restricted to particular tissues or disease states. The post-transcriptional layer is similarly underdeveloped. LncRNAs are typically short-lived, and their turnover by the nuclear and cytoplasmic surveillance machinery is what really sets their steady-state abundance, but this is rarely the focus of mechanistic studies^10^. Studies that work out the upstream regulatory architecture of an lncRNA, even when they say less about downstream targets, would help to fill this gap.

## Section 2: Biogenesis and host gene relationship of circular RNAs

Almost every circRNA paper asks what the circRNA does. Very few ask why the back-splicing event happened in the first place. The answer involves cis elements such as flanking inverted repeats and intron length asymmetry, together with a growing set of trans-acting RNA-binding proteins^11^. The catalogue is far from complete. Biogenesis is also highly context dependent. Stresses such as hypoxia, DNA damage, and immune activation reshape the back-splicing landscape, often in dramatic ways, and the mechanisms behind those shifts are mostly unknown. The relationship between a circRNA and its host gene is a second open question. We rarely know whether the two are coupled or independent, whether circRNA production comes at the expense of linear mRNA from the same locus, or whether the circRNA feeds back on its own host. The same set of questions applies to divergent, antisense and intronic lncRNAs, where it is often unclear whether the act of transcription, the RNA product, or both, carries the function.

## Section 3: Structure and function

Protein biochemistry accepted long ago that mechanism cannot be claimed without structure. Research on lncRNAs and circRNAs has been slower to adopt this standard. Most papers on lncRNA and circRNA mechanisms infer function from sequence and binding partners, with no information about the secondary or tertiary structure that actually supports those interactions. The tools have been available for years. In-cell chemical probing methods now map RNA structure at nucleotide resolution across the transcriptome^12^. Cryogenic electron microscopy of ribonucleoprotein particles is now routine for many complexes. Computational structure prediction has improved enough to be a useful starting point. Studies that map the structural modules of an lncRNA or circRNA identify the elements that drive its protein interactions, and test how RNA modifications change folding will move the field towards a genuine mechanism. They will also establish the foundation that any RNA-targeted therapeutic effort will eventually need.

## Section 4: Quantitative biology

Mechanism is a quantitative claim. A proposed sponge or scaffold function requires the regulator to be present at a concentration high enough to engage its targets. Single-molecule fluorescence in situ hybridization and absolute quantification studies have shown that roughly 40% of lncRNAs are present at ten or fewer copies per cell^7^. Most canonical sponge or scaffold mechanisms cannot operate at those concentrations. Reporting copy number, half-life and the stoichiometric plausibility of a proposed mechanism would strengthen such studies considerably. The same level of quantitative rigour applied for years in studies of transcription factors and signalling pathways could valuably be extended to lncRNA and circRNA research.

## Section 5: Isoform identity

A single lncRNA locus often produces several isoforms with different ends, splice patterns and polyadenylation sites. A single circRNA host gene can give rise to dozens of distinct circular products. The literature still routinely refers to a given lncRNA or circRNA as if it were a single, uniform molecule. Defining which isoform actually does the work, in which cell type and under which conditions, should be treated as a prerequisite for a mechanistic claim, not as an optional refinement. Long-read sequencing, isoform-specific perturbation and isoform-resolved expression analysis have made this practical.

The pattern at EMM mirrors the broader literature. Among 35 lncRNA and circRNA research papers we examined in EMM over the past decade (Table 1), 15 (43%) proposed ceRNA or miRNA sponging mechanisms and another 16 (46%) described lncRNA–protein binding events characterized primarily through pull-down and luciferase assays. Only one paper addressed any of the five areas highlighted above, and not a single paper in this period made biogenesis, RNA structure, absolute quantification or isoform-specific function a primary mechanistic claim.

Our argument is not purely a call for a cleaner mechanism. Each of the five areas above becomes meaningful when it informs how disease arises and how it might be treated. How does the transcription factor landscape at an lncRNA promoter change in disease tissue? Does the back-splicing programme of a host gene shift in patient samples, and does that shift carry diagnostic or prognostic information? Which structural elements of a disease-relevant lncRNA can be targeted by small molecules or antisense oligonucleotides? Are the copy numbers and isoform usage of a candidate therapeutic target consistent with the proposed mechanism of action? Studies that take these next steps, connecting intrinsic biology to disease and to therapy, will be of particular interest.

## A shift in emphasis

None of this implies that classical ceRNA and descriptive lncRNA-protein interaction studies no longer have a place. Rather, we suggest that such studies are most valuable when they meet a higher bar than has often been applied. A proposed mechanism is more convincing when it is quantitatively plausible, not merely consistent with what is already known. Binding interactions are more compelling when shown to be direct rather than inferred from prediction tools alone. As multi-system validation across cell lines, animal models and patient samples becomes a routine starting point rather than a main contribution, the underlying mechanism itself increasingly becomes what distinguishes one study from another. We hope that work taking up any of the directions sketched above, or other emerging directions aligned with this shift towards the lncRNA or circRNA itself, will help to shape where the field goes next. The broader point is simply that authors might ask what makes their work a substantive advance over the existing literature, rather than a familiar story with a new RNA name attached.
